# A Clinical Safety Assessment of Hybrid Fractional Laser Use at Increased Depths for Facial Skin Rejuvenation in Patients Undergoing Rhytidectomy

**DOI:** 10.1093/asjof/ojaf114

**Published:** 2025-09-16

**Authors:** Skylar J Sirmans, Maheen F Akhter, Michael Hernandez, Taylor Chishom, Nick Woltjen, Stephanie Bieber, Roxanne Engel, Edgar Sosa, Rahim S Nazerali, Alan J Durkin

## Abstract

**Background:**

Hybrid fractional lasers (HFLs) have revolutionized facial laser resurfacing treatments with their dual ablative and non-ablative wavelength technology. Although HFL therapy shows promising results, safety at skin depths beyond 150 μm is debated, particularly for its non-ablative wavelength. Some suggest deeper HFL delivery can enhance skin remodeling.

**Objectives:**

To assess the clinical safety of delivering HFL therapy at increased dermal depths up to 425 μm to induce collagen production and improve skin quality in patients undergoing rhytidectomy, and to determine whether this combination increases postoperative complications.

**Methods:**

A retrospective review (2017-2022) was conducted on patients undergoing rhytidectomy with or without concurrent intraoperative HFL therapy. Data on demographics, HFL parameters, and postoperative complications were collected and analyzed using descriptive statistics.

**Results:**

Of 169 patients (94.7% female, 97.0% Caucasian, average age: 63.3 years, Fitzpatrick score: 2.7), 62.1% received HFL treatment and 37.9% were controls. Patient characteristics were similar between groups. The average non-ablative laser depth was 355 μm ± 25, with a maximum of 425 μm. In the 12 months post-rhytidectomy, 7.6% of the HFL group experienced complications vs 3.0% in the control group, a non-significant difference (*P* = .13). No known HFL complications (burns, skin breakdown, hypo- and hyperpigmentation) were observed.

**Conclusions:**

HFL therapy at depths up to 425 μm may be safe for facial photorejuvenation. Patients with Fitzpatrick skin Types I to IV experienced no adverse effects. Combining HFL with rhytidectomy did not increase complication rates compared to rhytidectomy alone. These findings expand safety parameters for HFL use, optimizing clinical outcomes and long-term success of laser skin resurfacing.

**Level of Evidence:**

3 (Therapeutic) 
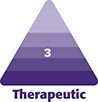

The advent of the world's first hybrid fractionated laser (HFL) marks a significant advancement in skin resurfacing technology. The hybrid feature uniquely combines ablative fractional laser (AFL; 2940 nm) and non-ablative fractional laser (NAFL; 1470 nm) wavelengths, simultaneously targeting both the surface and deep layers of the skin for effective rejuvenation.^1,2^ Designed with dual wavelengths, HFL therapy enhances both epidermal renewal and dermal collagen remodeling. It has shown histological and clinical promise in treating a range of esthetic concerns, including deep wrinkles, scarring, blemishes, and dyspigmentation. While multiple effective modalities exist for treating these conditions, including other laser and energy-based devices, recent studies suggest that the synergism of HFL's dual wavelengths may offer comparable or improved outcomes.^3,4^ Coupled with a reduced recovery time and favorable safety profile compared to full-field ablative lasers, the fractionated delivery method offers a promising option for patients seeking effective, yet minimally aggressive solutions for facial rejuvenation.^5-7^

One study on HFL therapy found that multiple low-to-moderate setting treatments, compared to a single high-setting session, resulted in greater patient satisfaction, shorter downtime, and fewer side effects, without compromising efficacy. This suggests a more balanced approach in terms of safety, tolerability, and clinical outcomes.^8^ According to a multi-expert panel, HFLs offer a high degree of treatment customization, allowing clinicians to effectively target pigmentation, texture irregularities, and mild wrinkling with minimal downtime and consistently favorable patient outcomes.^9^ Another study that evaluated HFL for facial photorejuvenation demonstrated a 100% satisfaction rate among 29 participants, with a high proportion reporting significant visible skin improvement. This study highlighted the procedure's favorable tolerability and efficacy in improving photodamaged skin and dyschromia, with minimal downtime and no serious adverse events.^10^

Beyond its cosmetic applications, HFL has also been used for broader medical applications, such as for the treatment of female urinary incontinence and menopause symptoms, due to its positive effects on epithelial thickness, reinforcing collagen, and enhancing vascularity.^11-13^

Comparatively, many studies on other laser technologies, such as fractional CO_2_ and Nd:YAG lasers, have demonstrated their safety and effectiveness for various skin conditions while showing minimal side effects and high patient satisfaction.^2,14,15^

Despite the growing popularity in the use of HFL for photorejuvenation, there is a notable gap in research regarding the safety and efficacy of using HFL at penetration depths >250 μm in the facial region. Although prior studies suggest that deeper penetration levels may enhance skin remodeling and rejuvenation, this hypothesis remains insufficiently validated in clinical settings.^16-18^ A closer look at patient outcomes and post-treatment complications after HFL resurfacing is warranted to understand whether lasering at increased depths is both safe and appropriate. This retrospective study seeks to bridge this knowledge gap by assessing the safety profile of the HFL when applied at depths of up to 425 μm on the face and neck status-post rhytidectomy, and we aim to provide empirical evidence that may enhance its utility in clinical practice.

## METHODS

A retrospective review was conducted at a single, private practice plastic surgery clinic. From June 2017 to May 2022, all patients aged 18 years and older who underwent primary or secondary rhytidectomy for the purpose of facial rejuvenation were included. The surgical technique of rhytidectomy varied among patients, and many received additional esthetic procedures, such as blepharoplasty, dermal filler injection, and structural fat grafting at the time of rhytidectomy. Patients who received intraoperative HFL therapy to the face and/or neck immediately after rhytidectomy for additional facial rejuvenation were categorized into the intervention group. Primary indications for HFL therapy included patients with visible signs of photoaging who sought additional cosmetic improvement of fine lines and textural skin irregularities, aiming to enhance overall skin quality in addition to surgical correction. Patients in the control group either declined laser resurfacing or did not express interest in addressing surface-level skin concerns beyond surgery. This study assesses the use of HFL therapy only in patients undergoing concurrent rhytidectomy. Patients who received HFL therapy as a standalone treatment were not included in the analysis. Those who did not receive any intraoperative laser skin resurfacing after rhytidectomy were categorized as control subjects. Patients who received any other laser resurfacing therapy besides the HFL, such as an ablative laser alone, were excluded from the study. Data was collected on patients' demographics, comorbidities (including smoking status), previous and concurrent surgical and non-surgical esthetic procedures, depth and coverage of HFL delivery, as well as any complications that occurred in the 12 months following rhytidectomy. All included patients had a minimum follow-up period of 12 months. Complications were considered major if they required readmission or reoperation, and minor if they self-resolved or were treated in the outpatient setting.

Patients are assessed preoperatively for a history of, or current use of, tobacco and/or electronic cigarets, and are counseled on smoking cessation. Patients are advised to stop smoking at least 4 to 8 weeks prior to their procedure. Patients with a history of herpes simplex virus or cold sores were prescribed prophylactic antiviral medications (acyclovir) 1 week prior to the procedure.

### Laser Procedure

Patients in the treatment group consented to resurfacing with the Sciton HALO Hybrid Fractional Laser (Sciton Inc., Palo Alto, CA), which simultaneously delivers NAFL (1470 nm) and AFL (2940 nm Er:YAG) fractional wavelengths in a single treatment. The treatment parameters were customized for each patient within the following ranges: NAFL (1470 nm) wavelength 100 to 700 µm depth, 5% to 30% density; AFL (2940 nm) wavelength 20 to 100 µm depth, 5% to 30% density. Patients received HFL therapy after undergoing preoperative screening for contraindications and receiving counseling on its risks, benefits, and complications. Upon completion of the rhytidectomy procedure, intraoperatively, while under general anesthesia, patients were treated with the HFL on the face and/or neck (Supplemental Figure 1). Appropriate eye protection was provided for the patient intraoperatively. HFL was delivered to 5 zones of the face: left and right mid-face and lower face, left and right forehead, and nose, including the glabella area (Supplemental Figure 2).^19^ To avoid compromising the vascular integrity of the rhytidectomy skin flap, the laser treatment was not applied over or across the edge of the skin flap. The treatment area was carried up to and stopped within 2 to 3 mm of the flap margin. Upon HFL delivery, laser coverage within each zone was recorded as a fraction of the total zone area. Depth of laser delivery was also recorded for each zone. This process was performed for both the AFL and NAFL wavelengths of the HFL. Mild erythema and/or punctate bleeding served as clinical endpoints of therapy. If bleeding was persistent after the procedure, gauze soaked with a mixture of epinephrine and water was applied to the lasered areas. Patients were given aftercare instructions and were advised to allow 7 to 14 days for recovery. Additional aftercare instructions included the use of broad-spectrum sunscreen (SPF 50 or higher), maintaining proper hydration, and strict avoidance of direct sunlight to prevent complications and support optimal healing. A hydrating cream and a gentle, non-active, fragrance-free cleanser were provided and recommended to minimize irritation. Patients were instructed to avoid touching, picking, or rubbing the treated area to reduce the risk of infection or scarring. The use of makeup was discouraged until suture removal to further decrease the risk of infection and irritation. Reintroduction of active skincare products (eg, retinoids, acids, vitamin C) should be postponed until the skin barrier has fully re-epithelialized and post-rhytidectomy recovery is complete.

### Statistical Analysis

Descriptive statistics were applied to compare outcomes between groups. Statistical significance was set at an alpha value of .05. A *χ*^2^ test, a 2-tailed Fisher's exact test, and an independent *t* test were used to determine if the differences were statistically significant. All data analysis was performed using Microsoft Excel (Microsoft Corporation, Redmond, WA), and data was stored on a restricted-access research folder.^20^ This retrospective study was conducted in accordance with the principles of the Declaration of Helsinki. All patients consented to the use and analysis of their data in this study.

## RESULTS

### Patient Characteristics

A total of 169 patients were included, with 62.1% (*n* = 105) patients in the treatment group and 37.9% (*n* = 64) patients in the control group. Most participants were female (94.7%) and Caucasian (97.0%), with an average age of 63.3 ± 7.5 years. Fitzpatrick skin Types I to IV were represented among the patient population, with a mean Fitzpatrick score of 2.7 ± 0.7. The subjects in the intervention and control groups were demographically very similar, as detailed in Table 1.

**Table 1.** Patient Demographics and Outcomes

	All patients (*n* = 169)	HFL (*n* = 105)	Control (*n* = 64)	*P*-value
Gender				.26
Male	5.3%	3.8%	7.8%	
Female	94.7%	96.2%	92.2%	
Age (mean ± SD)	63.3 ± 7.5	62.7 ± 7.3	64.2 ± 7.8	.19
Race				.36
Caucasian	97.0%	98.1%	95.3%	
Other/mixed	3.0%	1.9%	4.7%	
BMI (mean ± SD)	23.7 ± 4.0	23.8 ± 3.9	23.9 ± 3.1	.81
Fitzpatrick score	2.7 ± 0.7	2.7 ± 0.7	2.7 ± 0.7	.70
Type I	1.2%	1.0%	1.6%	.72
Type II	45.0%	48.6%	39.1%	.37
Type III	40.8%	35.2%	50.0%	.15
Type IV	13.0%	15.2%	9.4%	.31
Comorbidities				
Smoking history	28.7%	30.5%	26.6%	.59
Hypertension	18.9%	14.6%	25.8%	.05^a^
Diabetes	1.2%	1.0%	1.6%	.72
Previous rhytidectomy	17.0%	16.2%	15.6%	.92
Concurrent additional cosmetic surgery	57.8%	58.1%	57.5%	.95

HFL, hybrid fractional laser; SD, standard deviation; a, denotes statistical significance at the level of *P* < .05.

The study population included participants with 3 comorbid conditions: smoking history, hypertension, and diabetes mellitus. The most prevalent comorbidity was smoking history (28.7%), followed by hypertension (18.9%) and diabetes mellitus (1.2%). Additionally, (38.5%) of participants had at least one comorbidity, while (5.3%) had multiple comorbidities. In the Control group, there were significantly more patients with hypertension than in the HFL group (*P* = .05). However, most of the population did not have any significant comorbidities (56.8%) The distribution of comorbidities is summarized in Table 1.

Among the patients, 17% had undergone a previous rhytidectomy (secondary rhytidectomy) procedure. In addition to the current rhytidectomy, 57.9% of patients received concurrent additional cosmetic surgeries. The most common was an upper lid blepharoplasty. Supplemental Table 1.

### Laser Treatment Delivery

Patients in the intervention group received a single treatment of HFL therapy to the face immediately following rhytidectomy. The mean depth of delivery for the NAFL was 355 ± 25 μm across all areas of the face, with a minimum depth of 300 μm, and the maximum depth observed was 425 μm (Table 2). On average, the percent coverage per zone for the NAFL was 26 ± 5%, with a maximum of 34.9%. With the AFL component, the mean depth of delivery was 36 ± 21 μm across all areas of the face, with a minimum depth of 20 μm, and the maximum depth observed was 100 μm. The average percent coverage per zone for the AFL was 20 ± 3% with a maximum of 27.8%. The range of depth treated was 125 μm for NAFL and 80 μm for AFL.

**Table 2.** Mean HFL Depth and Coverage

	Face		Neck	
	Depth (µm)	Coverage (%)	Depth (µm)	Coverage (%)
Non-ablative wavelength	355 ± 25	26 ± 5	354 ± 37	16 ± 2.6
Ablative wavelength	36 ± 21	20 ± 3	21 ± 3	17 ± 2.3

HFL, hybrid fractional laser.

Additionally, 62.8% of the HFL patients also underwent photorejuvenation to the neck region. The average depth of the NAFL throughout the neck was 354 ± 37 μm with a minimum depth of 300 μm, and a maximum depth of 400 μm. The NAFL coverage of the neck was 16 ± 2.6% with a maximum of 23.2%. For the AFL component, a mean depth of 21 ± 3 μm was observed throughout the neck, with a minimum depth of 20 μm, and a maximum depth of 30 μm. The average percent coverage for the AFL was 17 ± 2.3% with a maximum of 22.3%.

### Postoperative Complications

**Figure 1.**
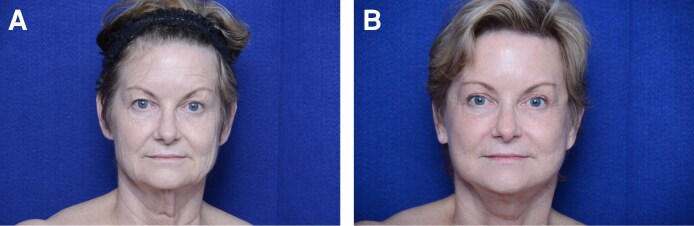
Sixty-seven-year-old female patient from the control group who underwent a facelift without HFL therapy, shown (A) preoperatively and (B) 12 months postoperatively. HFL, hybrid fractional laser.

**Figure 2.**
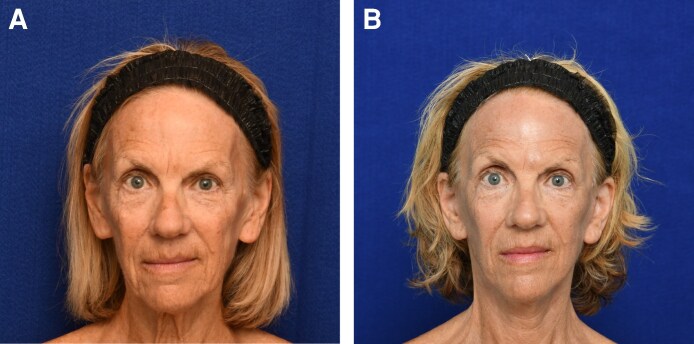
Sixty-eight-year-old female patient from the HFL group who underwent a facelift with HFL therapy, shown (A) preoperatively and (B) 12 months postoperatively. HFL, hybrid fractional laser.

A total of 10 (5.8%) patients experienced complications in the entire cohort, with 8 (7.6%) in the HFL group and 2 (3.0%) in the control group. There was no statistically significant difference in the rate of all-cause complications between groups (*P* = .13). A subgroup analysis indicates that HFL therapy does not significantly increase complication rates in patients undergoing either a primary or secondary rhytidectomy. While the overall complication rate for primary rhytidectomy was slightly higher in the HFL group (6.7%) compared to the control (3.1%), secondary rhytidectomy complications were lower overall, with rates of 1.0% in the HFL group and no complications observed in the control group. Statistical analysis revealed no significant differences between groups (*P* = .76 for HFL vs *P* = .51 for control; Table 3). Throughout the entire study sample, the most common complications were hematoma (1.8%), cellulitis (1.8%), and wound dehiscence (1.2%) (Table 4). The rates of each of these complications were very similar when comparing the HFL group to the control group (1.9% vs 1.5%, *P* = .87; 1.9% vs 1.5%, *P* = .87; 1.9% vs 0%, *P* = .53). Although less common, desquamation (0.6%) and epidermolysis (0.6%) were also observed, both of which occurred at a rate of 1.0% in the HFL group and 0% in the control group. Known complications of HFL, such as burns, skin breakdown, hypertrophic scarring, hypopigmentation, and hyperpigmentation, were not observed among any of the patients in the HFL group, nor in the overall study sample. Additionally, no skin loss or skin necrosis occurred in either of the groups (Figures 1, 2).

**Table 3.** Complications of Primary vs Secondary Rhytidectomy

	All patients (*n* = 10) (%)	HFL (*n* = 8) (%)	Control (*n* = 2)
Rhytidectomy type			
Primary	5.3	6.7	3.1%
Secondary	0.6	1.0	—
*P*-value	.58	.76	.51

HFL, hybrid fractional laser.

**Table 4.** Postoperative Complications Within 12 Months

	All patients (*n* = 169) (%)	HFL (*n* = 105) (%)	Control (*n* = 64)	*P*-value
Complications	5.8	7.6	3.0%	.13
Hematoma	1.8	1.9	1.5%	.87
Cellulitis	1.8	1.9	1.5%	.87
Wound dehiscence	1.2	1.9	—	.53
Desquamation	0.6	1.0	—	1.00
Epidermolysis	0.6	1.0	—	1.00

HFL, hybrid fractional laser.

## DISCUSSION

The objective of our study was to evaluate the safety profile and efficacy of a single treatment of HFL therapy at increased depths, up to 425 μm, in conjunction with patients undergoing a cervicofacial rhytidectomy procedure. Our findings demonstrated that this combined approach was safe, with no significant difference in complication rates between the HFL and control groups. The complications observed were primarily associated with the facelift procedure itself rather than the increased depth of the HFL treatment. These results align with prior work by Fusano et al, who conducted HFL treatments at comparable depths and reported a strong safety profile with minimal complications. Their study demonstrated that HFL treatments resulted in faster healing times and lower risk of pigmentary changes compared to traditional ablative CO_2_ lasers, reinforcing the safety of deep HFL resurfacing in Fitzpatrick skin Types II and III.^1,7,11^ Furthermore, the similarity in patient demographics and comorbidities, along with minimal variation in the lasering technique and operative details, suggests that HFL therapy at depths up to 425 μm into the dermis does not present any inherent safety concerns when performed on the face and neck. The absence of common HFL-associated adverse side effects, such as burns, scarring, hypo- or hyperpigmentation, or skin breakdown among our patient cohort reinforces this conclusion.^3,10,11^ However, even with a predominance of patients with lower Fitzpatrick skin types, which are generally less susceptible to laser-induced injury, no adverse effects or post-inflammatory hyperpigmentation (PIH) were observed, supporting the safety profile of deep HFL therapy up to 425 μm.^21^

Importantly, no instances or signs of threatened skin loss, recognized in our study, as any areas of impending necrosis, were observed in either group. This is a critical consideration given the partially de-vascularized flaps created during rhytidectomy, which are more vulnerable to additional thermal or mechanical insult.^21^ However, our results suggest that, when properly applied, deep HFL does not compromise skin viability or impair healing in these at-risk regions.

Complications such as hematoma, cellulitis, and wound dehiscence were evenly distributed between the HFL and control groups, supporting the conclusion that these issues are distinctive of the rhytidectomy procedure and not exacerbated by the addition of deep HFL therapy.^22^

Statistical analysis revealed no significant differences between groups (*P* = .76 for HFL vs *P* = .51 for control), indicating that complication rates did not differ substantially between primary and secondary procedures and were comparable across groups, supporting the safety of HFL in both settings. Most complications occurred within 1 week (within 7 days) of the procedure and followed the expected postoperative recovery course. The most common complications observed in both groups were post-auricular hematoma and cellulitis, occurring in a 2-to-1 ratio in the HFL and control groups, respectively. Notably, neither of these complications is commonly associated with HFL therapy. These findings are consistent with previously reported complication profiles in standard rhytidectomy cases, where hematoma is typically the most frequent early complication, followed by cellulitis.^23^

In the HFL group, 3 distinct complications were observed; however, none were attributable to the HFL treatment itself. One patient experienced a localized spontaneous wound dehiscence at the post-auricular margin, which was attributed to a difficult staple removal at the 2-week follow-up visit. Another patient developed minor epidermal desquamation also at the post-auricular margin, determined to be caused by the patient wearing eyeglasses underneath the compression bandeau, resulting in irritation and minor desquamation to the area. Importantly, this occurred outside the HFL treatment zone. All incisions remained intact with no signs of infection. One patient, in direct contradiction to postoperative care instructions, persistently smoked marijuana and subsequently developed bilateral post-auricular epidermolysis. The patient was treated with wound care, bacitracin, and smoking cessation counseling. Importantly, none of these complications significantly delayed wound healing or impacted the anticipated rhytidectomy and HFL postoperative recovery course. These isolated events underscore the importance of distinguishing complications resulting from surgical technique or postoperative care from those directly attributable to HFL therapy.

Combining deep HFL therapy with rhytidectomy is not only safe but potentially more efficacious. This combination allows for improved facial rejuvenation that rhytidectomy alone cannot provide. While rhytidectomy effectively addresses skin laxity and facial ptosis, it does not treat surface-level issues such as fine lines, pigmentation irregularities, collagen loss, or textural changes. Deep HFL therapy promotes neocollagenesis and dermal remodeling, complementing the lifting effects of surgery by improving overall skin tone and quality and effectively targeting the physiological changes that occur with aging and photodamage.^3,4,10,11,16,17^ The use of HFL immediately post-rhytidectomy permits both physiological and manual enhancement, potentially extending the longevity of the esthetic rejuvenation effects by maximizing the benefits of each treatment modality; this combined approach may lead to improved overall outcomes.^22,24,25^ Given that a portion of our patients were undergoing secondary facelifts, it is clear that facial rejuvenation requires maintenance and intensive therapy. Incorporating HFL in the immediate postoperative period may contribute to more durable long-term outcomes; however, further studies are needed to confirm this potential benefit.

To mitigate the risk of complications, it is essential to identify the patient's skin type, comorbid conditions, Fitzpatrick score, and specific concerns. Once these characteristics are established, the HFL treatment parameters can be adjusted accordingly and personalized for each patient. Patients with darker, non-Caucasian skin types are at an increased risk of post-procedural pigmentary changes, including PIH and scarring. Accurate assessment of Fitzpatrick skin types is important, as it informs therapeutic decisions.^21,26^ However, these patients can still safely undergo deep NAFL therapy by reducing the treatment density. This adjustment minimizes the risk of adverse effects while ensuring optimal dermal remodeling at increased depths.

Our HFL cohort included Fitzpatrick skin Types I to IV, the majority being Type II and Type III, consisting of 51 (48.6%) and 37 (35.2%) patients, respectively. There was only 1 Type I and 16 Type IV individuals. In the Type I patient, the depth of the NAFL was 350 μm with 15% density and 20 μm with 18% for the AFL. The Type IV individuals had a mean NAFL depth of 338 μm at 25% density, with a maximum depth of 400 μm, and for the AFL treatment, the mean depth was 41 μm with 20% density. No complications were observed in the Type I patient. A single Type IV patient experienced a post-auricular hematoma; however, this complication was not related to the HFL treatment. Although Type IV individuals are generally more susceptible to laser-induced adverse effects,^21,27^ none of the 16 patients incurred a laser-induced injury, and no other complications were observed in the other 15 Type IV patients within the 12-month follow-up period. These findings demonstrate the safety of deep HFL at the specified parameters in Fitzpatrick skin classifications II to IV.

### Limitations

Although our study provided promising insights, several limitations must be acknowledged. First, our predominantly Caucasian female patient population with Fitzpatrick skin Types II and III limits the generalizability of our results, as only 1 individual had Type I skin and 16 had Type IV. Future research should include a broader range of skin types and patients with varied comorbidities to better assess HFL therapy's safety across diverse groups. Additionally, the low complication rates in both treatment and control groups limited our ability to conduct extensive statistical analyses. A larger and more diverse sample would allow for a more detailed examination of potential complications. Another limitation of our study is the unequal matching of subjects between the treatment and control groups. The treatment group included nearly twice as many participants as the control group. This imbalance may have introduced potential biases in outcome comparisons and limited the statistical power of between-group analyses. Further, this study is subject to selection bias, as the providers used their clinical judgment to determine laser treatment candidates, which may further limit the generalizability of the findings.

## CONCLUSIONS

This study evaluated a demographically similar group of patients undergoing a primary or secondary rhytidectomy, with and without adjunctive HFL therapy for facial rejuvenation, including skin Types I to IV in a generally healthy patient population without any significant comorbidities. Patients in the treatment group received HFL to the face and/or neck at mean depths up to 425 μm. There was no statistically significant difference in complication rates between the HFL and control groups. The most common complications observed were hematoma, cellulitis, and wound dehiscence, which occurred at comparable rates between groups. No cases of skin loss, necrosis, burns, hypertrophic scarring, hypo- or hyperpigmentation were observed in either group. These findings support the objective of our study, which was to assess the safety of delivering HFL therapy at increased depths in combination with surgical rhytidectomy to improve facial rejuvenation outcomes.

## Supplemental Material

This article contains supplemental material located online at https://doi.org/10.1093/asjof/ojaf114.

## Supplementary Material

ojaf114_Supplementary_Data
