# Supplementary material for: A Clinical Safety Assessment of Hybrid Fractional Laser Use at Increased Depths for Facial Skin Rejuvenation in Patients Undergoing Rhytidectomy
Source: Aesthet Surg J Open Forum. 2025 Sep 16;7:ojaf114. doi: 10.1093/asjof/ojaf114 (PMC12586331; doi:10.1093/asjof/ojaf114)
Supplement: ojaf114_Supplementary_Data [file ojaf114_supplementary_data.zip › Supplemental Table 1 updated 7.13.25.docx]

| **Supplemental Table 1.** |  |  |
| --- | --- | --- |
|  |  |  |
| **List of Additional Concurrent Surgical and Non-surgical Procedures** | | |
| **Surgical** | **Non-Surgical** | |
| ﻿Abdominoplasty (HFL - 2, Control - 1) | Belotero® | |
| ﻿Blepharoplasty (HFL - 36, Control - 20) | Juvederm® | |
| ﻿Breast augmentation (HFL - 2, Control - 2) | Platelet-Rich Plasma | |
| Breast implant exchange (HFL - 4, Control - 3) | Radiesse® | |
| Breast reduction (HFL - 1, Control - 2) | Restylane® | |
| Canthoplasty (HFL - 9, Control - 10) | Revanesse® | |
| ﻿Capsulectomy (HFL - 4, Control - 3) | Xeomin® | |
| ﻿Genioplasty (HFL - 4, Control - 2) |  | |
| Liposuction (HFL - 4, Control - 5) |  |  |
| Mastopexy (HFL - 5, Control - 1) |  |  |
| Rhinoplasty (HFL - 1, Control - 1) |  |  |
| Scar revision (HFL - 1, Control - 0) |  |  |
| Structural Fat grafting (HFL - 13, Control - 18) |  |  |
| Trichophytic Browlift (HFL - 12, Control - 3) |  |  |

* Belotero® – Merz Aesthetics, Raleigh, NC, USA

Juvederm® – Allergan, Inc., an AbbVie company, Irvine, CA, USA

Radiesse® – Merz Aesthetics, Raleigh, NC, USA

Restylane® – Galderma Laboratories, Fort Worth, TX, USA

Revanesse® – Prollenium Medical Technologies Inc., Aurora, Ontario, Canada

Xeomin® – Merz Aesthetics, Raleigh, NC, USA
