# Supplementary figures and images for: A Clinical Safety Assessment of Hybrid Fractional Laser Use at Increased Depths for Facial Skin Rejuvenation in Patients Undergoing Rhytidectomy
Source: Aesthet Surg J Open Forum. 2025 Sep 16;7:ojaf114. doi: 10.1093/asjof/ojaf114 (PMC12586331; doi:10.1093/asjof/ojaf114)

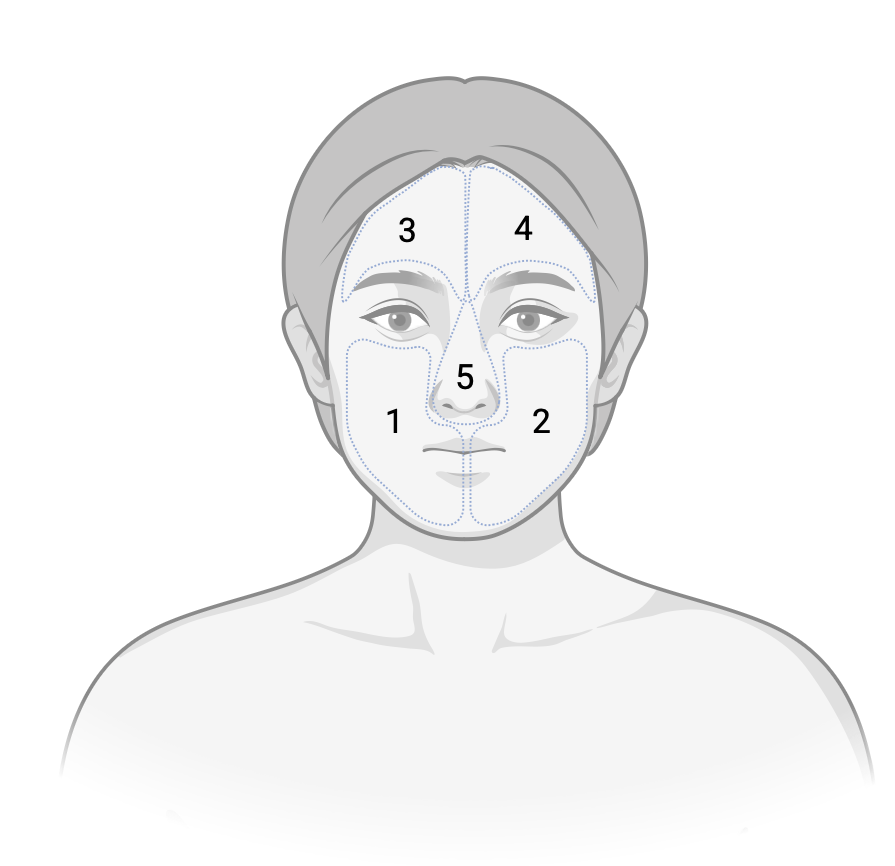

Supplement: ojaf114_Supplementary_Data [file ojaf114_supplementary_data.zip › Supplemental Figure 2. Anatomic Illustration of Hybrid Fractional Laser (HFL) treatment zones 1-5 of the face.png]

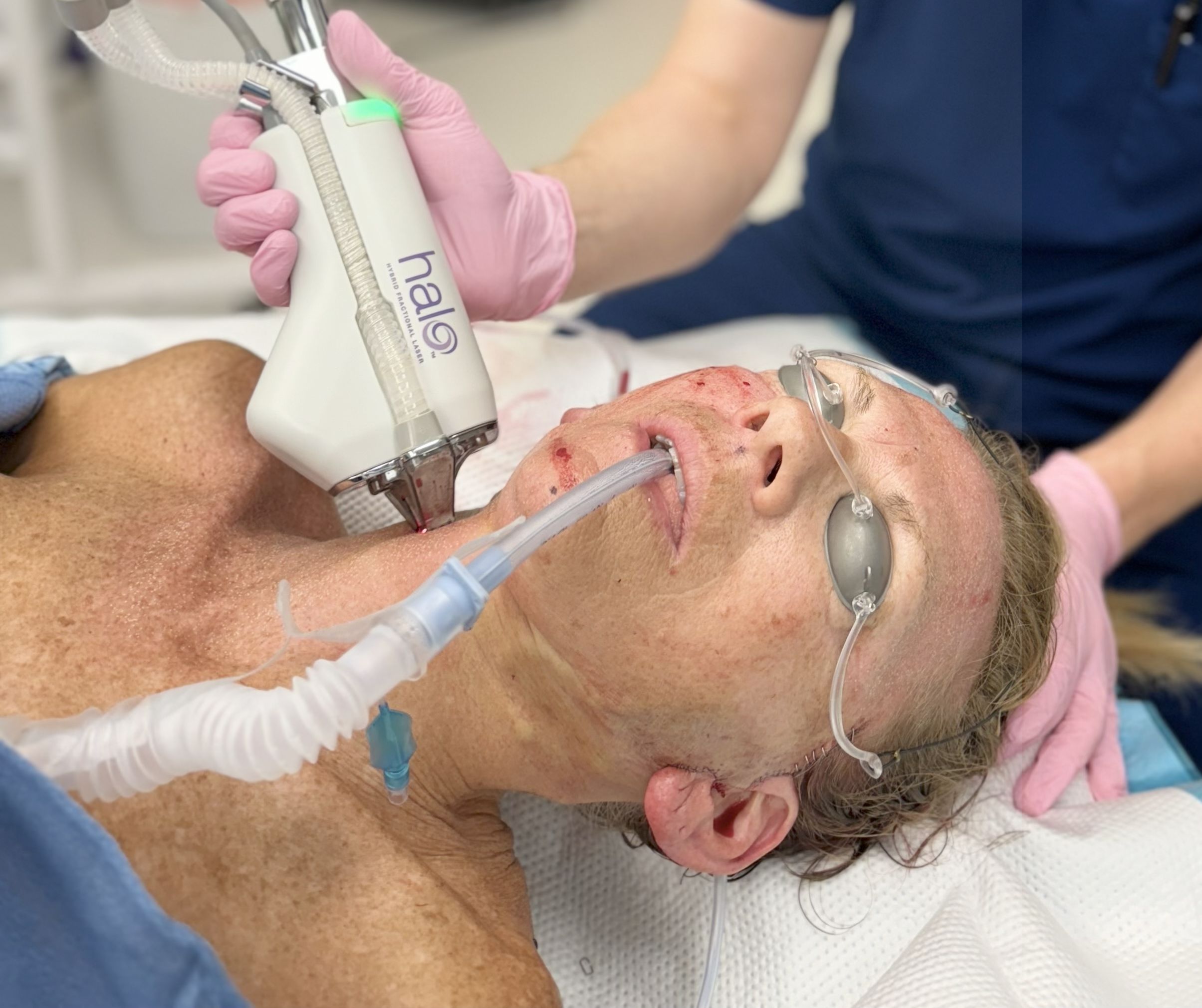

Supplement: ojaf114_Supplementary_Data [file ojaf114_supplementary_data.zip › Supplemental_Figure_1.jpg]
